# Patient-derived cell-based pharmacogenomic assessment to unveil underlying resistance mechanisms and novel therapeutics for advanced lung cancer

**DOI:** 10.1186/s13046-023-02606-3

**Published:** 2023-01-30

**Authors:** Namhee Yu, Mihwa Hwang, Youngjoo Lee, Bo Ram Song, Eun Hye Kang, Hanna Sim, Beung-Chul Ahn, Kum Hui Hwang, Jihyun Kim, Sehwa Hong, Sunshin Kim, Charny Park, Ji-Youn Han

**Affiliations:** 1 Research Institute, National Cancer Center, Goyang-si, Gyeonggi-do 10408 Republic of Korea; 2 Department of Precision Medicine, National Institute of Health, Korea Disease Control and Prevention Agency, Cheongju, 28159 Republic of Korea

**Keywords:** Patient-derived cell, Pharmacogenomics, Small-cell lung cancer, EGFR-TKI, Osimertinib resistance, YAP/TAZ-AXL axis

## Abstract

**Background:**

A pharmacogenomic platform using patient-derived cells (PDCs) was established to identify the underlying resistance mechanisms and tailored treatment for patients with advanced or refractory lung cancer.

**Methods:**

Drug sensitivity screening and multi-omics datasets were acquired from lung cancer PDCs (*n* = 102). Integrative analysis was performed to explore drug candidates according to genetic variants, gene expression, and clinical profiles.

**Results:**

PDCs had genomic characteristics resembled with those of solid lung cancer tissues. PDC molecular subtyping classified patients into four groups: (1) inflammatory, (2) epithelial-to-mesenchymal transition (EMT)-like, (3) stemness, and (4) epithelial growth factor receptor (*EGFR*)-dominant. *EGFR* mutations of the EMT-like subtype were associated with a reduced response to EGFR-tyrosine kinase inhibitor therapy. Moreover, although *RB1*/*TP53* mutations were significantly enriched in small-cell lung cancer (SCLC) PDCs, they were also present in non-SCLC PDCs. In contrast to its effect in the cell lines, alpelisib (a PI3K-AKT inhibitor) significantly inhibited both *RB1*/*TP53* expression and SCLC cell growth in our PDC model. Furthermore, cell cycle inhibitors could effectively target SCLC cells. Finally, the upregulation of transforming growth factor-β expression and the YAP/TAZ pathway was observed in osimertinib-resistant PDCs, predisposing them to the EMT-like subtype. Our platform selected XAV939 (a WNT-TNKS-β-catenin inhibitor) for the treatment of osimertinib-resistant PDCs. Using an in vitro model, we further demonstrated that acquisition of osimertinib resistance enhances invasive characteristics and EMT, upregulates the YAP/TAZ-AXL axis, and increases the sensitivity of cancer cells to XAV939.

**Conclusions:**

Our PDC models recapitulated the molecular characteristics of lung cancer, and pharmacogenomics analysis provided plausible therapeutic candidates.

**Supplementary Information:**

The online version contains supplementary material available at 10.1186/s13046-023-02606-3.

## Background

Lung cancer is the leading cause of cancer-related mortality worldwide [1]. The development of targeted therapies, such as epidermal growth factor receptor (EGFR)-tyrosine kinase inhibitors (TKIs), have helped to extend the survival time of patients; however, the improvement in progression-free survival eventually fails in cases of advanced lung cancer owing to resistance development [2]. Thus, treatment tailored to overcome resistance is need to improve prognosis. To develop novel therapeutics, several cellular, organoid, and mouse models are available for use in pharmacological platforms [3–5]. Although organoid and mouse models recapitulate the heterogeneous molecular characteristics of patient biopsies, their establishment is labor-intensive and they have relatively low tumor-formation rates [3]. Models based on patient-derived immortalized cells provide good reproducibility to the cohort, but have less heterogeneity, are more mesenchymal in nature, and have distinct chemical and genetic dependencies [6]. Fortunately, models derived from short-term–cultured patient-derived cells (PDCs) retain the genomic characteristics of solid tumor biopsy better than immortalized cell lines [3, 7, 8]. Thus, the use of PDC models can help to enlarge the scope of drug screening to include more chemicals and multiple doses. To date, PDC pharmacogenomic platforms have been successfully used in treating glioblastoma and gynecologic and gastric cancers [3, 7, 8]. A PDC platform was also established to screen drugs for non-small cell lung cancer (NSCLC) [9]. However, this platform required cell culture for 2–6 months and demonstrated a 50% success rate. These numbers represent an obstacle for its application in the medical field to fill the need of immediate patient-tailored drug prediction. Therefore, establishing a PDC platform for refractory lung cancers would satisfy the clinically unmet needs of therapeutic and drug-resistance research.

Advances in large-scale lung cancer genomics approaches have helped to tackle the challenges posed by tumor heterogeneity, therapeutic evolution, and histology [10–12]. EGFR-TKI therapies possess good *EGFR*-mutation selectivity, but resistance occasionally evolves via activation of non-targetable bypass pathways such as neuronal differentiation or *KRAS* amplification [11, 13, 14]. Treatment of small-cell lung cancer (SCLC) is associated with an additional challenge of the lack of further effective therapies in the face of rapid resistance acquisition to platinum-based chemotherapy [15]. Moreover, information available on driver genes and various (TKI-resistant) variants of refractory lung cancers have not been effectively translated into clinical targeted therapies.

To bridge this gap, in this study, we developed PDC models using mainly the pleural effusions of refractory lung cancer patients. Using our platform, we explored drug candidates and target regulatory mechanisms according to genomic features and lung cancer molecular types. We further assessed the pharmacogenomic characteristics of the cells by screening their drug responses and performing next-generation sequencing. To establish integrative analysis for drug and genomic characteristics, statistical and machine-learning methods were employed to investigate the sensitivity to each drug according to molecular subtype, cancer types, therapeutic groups, and variants. This PDC platform and associated analysis can highlight novel drug candidates and target pathways to improve the personalized treatment of advanced lung cancer and facilitate further research in this regard.

## Methods

### Lung cancer sample acquisition and PDC establishment

Cancer samples were collected from patients with advanced or refractory lung cancer diagnosed and treated at the National Cancer Center in Korea between December 2016 and February 2020. The histological types were determined according to the 2015 World Health Organization classification of lung tumors. This study was approved by the National Cancer Center Institutional Review Board (approval number NCC2019-0082). All patients provided written informed consent. PDC establishment and drug screening details are described in Additional file 1 (Supplementary methods).

### Drug sensitivity screening using PDCs and cell lines

Stabilized PDCs were seeded in 384-well plates (1000 cells/20 μl/well) in quadruplicate for each treatment. A total of 16 or 48 compounds were used for screening each PDC sample (Additional file 2: Table S1, S2). After overnight incubation, the cells were treated with one drug at a 5-fold serial dilution for a total of 6 doses (50 μM ~ 16 nM). Cell viability was measured after 72 hrs of treatment using the CellTiter-Glo Luminescent Cell Viability Assay kit (Promega, Madison, WI, USA) and an Infinite 200 Pro system (TECAN, Mannedorf, Switzerland). Each screening plate contained a dimethyl sulfoxide (DMSO)-only vehicle to calculate relative cell viability and normalize the data. Dose response curve (DRC) fitting and area under the curve (AUC) values were assessed using GraphPad Prism 5.3 (GraphPad Software Inc., San Diego, CA, USA). A screening compound library was newly prepared every month and tested for the preservation of chemical activities using NSCLC cancer cell lines (A549, PC9 and H1299). All library compounds were purchased from Selleckchem (Houston, TX, USA).

### Targeted next-generation sequencing (NGS) dataset and molecular subtype identification

Mutation and copy number variant (CNV) calling were performed using NGS and targeted sequencing. Gene expression profiles and fusion genes were identified from RNA-sequencing data.

To identify somatic mutations from target-seq data, we started with preprocessing with quality check, read trimming using Trimmomatic 0.39, and alignment using BWA 0.7.17 to hg19 [16, 17]. Alignment bam files were recalibrated and realigned for target regions using Picard 1.119 and GATK 4.1.3.0 [18]. Next, we called somatic mutations using Mutect2, referring to the panel-of-normal from the 1000 Genomes PON and gnomAD VCF files [18]. The somatic mutation results were annotated using Oncotator [19]. Additionally, we eliminated germline variants registered in the 1000 Genomes Project. The CNV profile was identified using GATK 4.0.4.0, and CNV peak calling was performed by GISTIC2 [20]. Additionally, we selected CNV genes as those with high amplification (log2 CNV > 2), high amplification rate (> 5%), and a correlation with mRNA expression (*P* value < 0.01). The tumor mutation burden (TMB) of the mutation count per mega base pair (perMbp) values was calculated by Maftools [21]. We classified TMB values into low (TMB < 0.1), middle (0.1 ≤ TMB < 0.2), and high (0.2 ≤ TMB) groups.

To additionally classify the functionality of the genes with the most recurrent mutations (*TP53* and *EGFR)*, we categorized previously reported hotspot and non-hotspot mutations. *TP53* mutations were classified into hotspot and non-hotspot mutations [22]. *EGFR* variants were divided into four types (TARGET: exon 19 deletion, L858R), T790M acquisition (T790M^aq^), not otherwise specified mutations (NOS), and NOS acquisition with target (NOS^aq^) [23]. The co-occurrence of mutated genes was tested by Fisher’s exact test for genes with recurrence > 3 and MutSig *P* value < 0.05 [24]. The double mutation pairs were referred for drug sensitivity test as well as single mutation cases.

RNA-seq analysis also proceeded with similar preprocessing with quality check, read trimming using Trimmomatic, and alignment using STAR v2.7.0a to hg19 referring to gene model ENSEMBL release 75 [16, 25]. The gene expression profile was extracted from RPKM quantified from bam files using RSEM v1.2.31 [26]. Fusion genes were identified from RNA-Seq and merged with the results from three callers, Defuse, PRADA v1.2 and STAR-Fusion v1.7.0 [25, 27, 28]. Fusions were annotated using Pegasus, and we extracted drug-targetable candidates involved in kinase or oncogenic signaling from published databases from TCGA and COSMIC [29–32].

To classify the RNA molecular subtype of the samples, we performed nonnegative matrix factorization (NMF) clustering using the RPKM gene expression profile. We assessed optimal cluster size from 2 to 5. Finally, cluster size for molecular subtype was chosen to computationally present a clear consensus plot (*n* = 4). To extract the transcriptomic characteristics of RNA subtypes, pathway activities for each sample were estimated using gene set variation analysis (GSVA) according to HALLMARK gene set collections [13]. Next, the difference in pathway activities according to RNA subtypes was tested by limma [33]. Additionally, the up-regulation of stemness-associated gene signatures were assessed using GSVA [34]. The gene signatures were collected from microarray, and ChIP-seq of two human embryonic stem cell gene sets, target genes of transcription factors (NANOG, OCT4, SOX2, and MYC), and Polycomb targets (Suz12, Eed, H2K27, and PRC2) to be under-expression to embryonic stem cells [35]. The difference of collected signature scores for subtype was tested by Wilcoxon rank-sum test.

### Comparison of PDCs with other lung cancer datasets

We evaluated the characteristics of PDCs and survival using external lung cancer cohorts. First, to evaluate the genomic concordance of PDCs with solid tumor biopsies, we assessed the similarity of mutations and expression profiles between our PDCs and The Cancer Genome Atlas (TCGA) lung adenocarcinoma (LUAD) datasets [10]. Before comparison, the transcriptome batch effect was eliminated using ComBat [36]. The expression profiles were verified from a principal component analysis plot. The frequencies of the most recurrent mutated genes in PDCs were also compared with those in TCGA dataset.

Next, prognostic significance according to distinct molecular subtypes determined based on gene expression profiles was evaluated using meta-transcriptome datasets: five National Center for Biotechnology Information Gene Expression Omnibus lung cancer datasets and TCGA-LUAD datasets (*n* = 1587 patients; see Additional file 3, Fig. S1). The batch effect among multiple datasets was also eliminated using ComBat. We respectively calculated the four subtype signature scores from the meta-transcriptome using GSVA to obtain differentially expressed gene (DEG) sets (*n* = 300). The subtype DEGs were acquired using the limma test from our PDCs. Next, the log-rank test and Cox proportional hazard analysis were performed to compare overall survival according to high (> 25%) and low (≤ 25%) scores for each subtype.

### Drug sensitivity test according to genomic variants and groups

To evaluate the drug sensitivity of our PDCs according to genomic characteristics, we performed tests for multiple conditions, including lung cancer histologic type, EGFR-TKI therapy group, mutations, CNVs, fusions, RNA subtypes, and co-occurring mutation pairs. *TP53* and *EGFR* mutations were additionally categorized (details are described in the Results). We performed the Wilcoxon rank-sum test using area under the dose response curve (AUC) values between the two groups. *P*-values were adjusted using the Benjamini–Hochberg method.

To compare alpelisib response of *RB1*/*TP53* cells between PDCs and cell lines, we additionally collected drug screening dataset of CCLE lung cancer cell lines (*n* = 70) to include SCLC (*n* = 7) [5]. Alpelisib sensitivity test was also performed by Wilcoxon rank-sum test for mutated cases, and SCLC lung cancer type.

### Evaluation of cell cycle inhibitors effective for SCLC and the associated gene FOXM1

We investigated the transcriptome characteristics of SCLC from differentially expressed gene (DEG) analysis to compare SCLC with NSCLC using limma [33]. Next, gene set enrichment analysis (GSEA) was performed using the upregulated DEGs for each group by referring to WikiPathways [37, 38] To evaluate drugs and genes for SCLC, we additionally investigated drug sensitivity data for SCLC versus NSCLC from cell lines. Our drug response screening result for AZD7762 was acquired from 13 NSCLC cell lines and 5 SCLC cell lines, like our PDCs. To assess the similarity of drug signature genes between cell line and PDC, we additionally calculated Pearson correlation coefficient [5].

The siRNAs targeting FOXM1 (Hs_FOXM1_6 and Hs_FOXM1_7) and the control siRNA were purchased from QIAGEN (Foster City, CA, USA). H69 and H209 cells were transiently transfected with siRNAs using a NEPA21 electroporator (NEPA GENE, Chiba, Japan). Suspension cells (1 × 10^6^) with 100 pmole siRNA in 100 μl OPTI MEM media per cuvette were subjected to electroporation with program No. 5 following the manufacturer’s instructions.

### Drug response-associated gene signature extracted using machine learning

To extract gene sets related to the response to each drug, we used the expression profiles to filter out genes according to mean expression level ≤ 1 and standard deviation ≤2. AUC values were transformed to a log_2_ scale, and gene expression values were converted to z scores of log_2_-scaled RPKM values. First, we selected the top 500 correlated genes with the AUC values for each drug. Next, we performed elastic net regularization to extract the gene feature importance for each drug response [3, 39]. The glmnet R package was applied to optimize parameters from nine values of α ∈ [0.1,0.9] and 50 values of λ ∈ [0.01,100] to minimize the root mean squared error using 10-fold cross-validation [40]. Bootstrapping was performed 500 times using the R boot package to extract drug gene signatures from features [8, 41]. Finally, gene feature importance was extracted for each drug, and genes were ranked to define drug signature gene sets. Next, we performed GSVA to investigate pathways enriched for each drug gene signature using Reactome with significant enrichment was assessed at *P* < 0.1 [34, 42].

### Molecular characteristics and drug identification according to EGFR-TKI therapeutics

To identify the molecular features related to EGFR-TKI therapeutics, we categorized therapeutic groups from 27 PDC samples. We categorized these patients into four groups (Fig. 5A): (1) BASELINE, PDCs acquired from *EGFR*-mutated patients that did not receive any treatment; (2) POST1, PDCs without *EGFR* T790M mutation, acquired after disease progression to the first-line use of first- or second-generation EGFR-TKIs; (3) POST2, PDCs with *EGFR* T790M, acquired from patients after first- or second-generation EGFR-TKI treatment; and (4) POST3, T790M-positive PDCs, acquired after disease progression to second-line use of the third-generation TKI osimertinib. To identify gene regulation according to these four treatment groups, we identified upregulated DEGs (*P* < 0.01) for each group and associated pathways using limma and GSEA (*P* < 0.1 )[38, 43]. Additionally, we collected known EGFR-TKI resistance pathway gene sets: MAPK, PI3K-AKT, JAK-STAT, Wnt β-catenin, plasminogen activation, neuroendocrine activation, YAP/TAZ, MET, HER2, RAS, ERK, KRAS, and TAM (TYRO3-AXL-MERTK) family genes [13, 14, 43–46]. Resistance pathway scores were calculated using GSVA for each PDC. The score difference of the four therapeutics groups was evaluated using the Wilcoxon rank-sum test [34].

When exploring sensitivity to drugs for each EGFR-TKI group, the Wilcoxon test was also performed to determine the AUC value difference. We additionally demonstrate our “sensitive-drug candidates” assessed from 27 PDCs extended to PDC pools (*n* = 70). To improve the statistical reliability of the current dataset, we also collected additional drug screening results of extended *EGFR*-mutated PDCs (*n* = 70) acquired from patients previously treated with EGFR-TKIs based only on the clinical treatment profile without the corresponding NGS profile.

The generation of osimertinib-resistant cell lines and experiments are described in Additional file 1 (Supplementary methods).

## Results

### Establishment and molecular characteristics of lung cancer PDCs

**Fig. 1:**
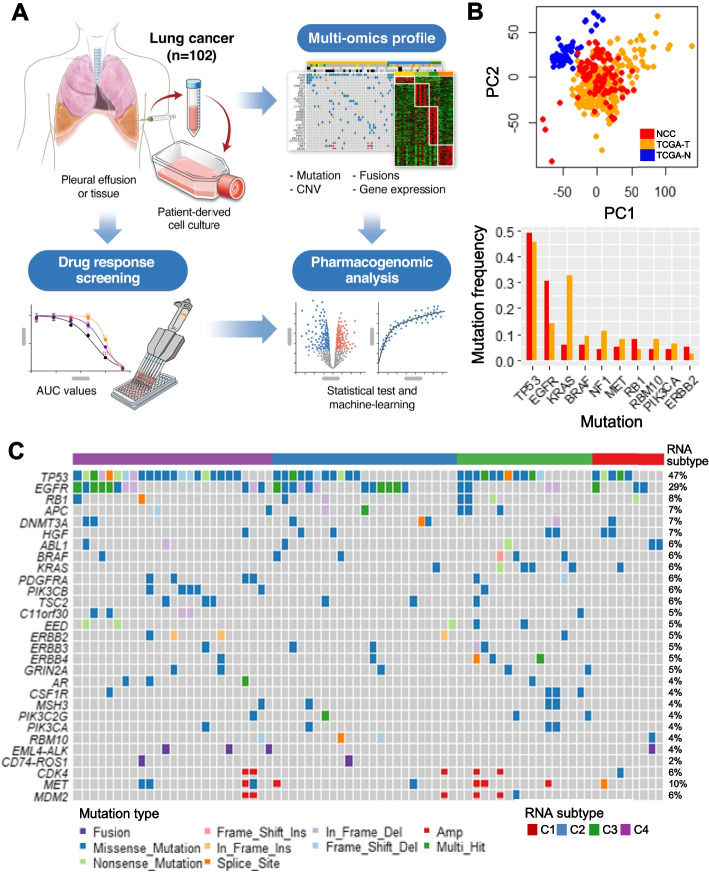
Genomic profiles of refractory lung cancer patient-derived cells (PDCs) for pharmacogenomic analysis. **A** Graphical workflow of pharmacogenomic analysis of lung cancer PDCs. **B** Gene expression and mutation profile comparison of our PDCs with TCGA samples. The scatterplot presents the principal component analysis for TCGA tumors, normal tissue samples, and PDCs. The bar plot indicates the mutation frequency of TCGA samples and PDCs. TCGA-T (orange) indicates tumor samples and TCGA-N (blue) indicates the adjacent normal tissue. **C** Genomic landscape heatmap of PDCs to harbor top-ranked somatic variants

We established a PDC collection of 102 samples (National Cancer Center; see Additional file 2, Table S1) from patients with advanced lung cancer enrolled in this study (Fig. 1A). PDCs were primarily collected from pleural effusions (92.2%), and secondarily from pericardial effusions (4.9%), ascites (2.0%), and tissues (1.0%; Additional file 2, Table S1). The pathologic type was adenocarcinoma (ADC; 84.3%), SCLC (5.9%), and miscellaneous types (squamous cell carcinoma, 4.9%; sarcomatoid carcinoma, 3.9%; and not otherwise specified, 1%). The drug panel used for response screening included 48 anti-cancer compounds of seven classes targeting angiogenesis (*n* = 3), the cell cycle (*n* = 8), DNA damage (*n* = 6), MAPK (*n* = 3), PI3K/AKT/mTOR (*n* = 3), protein tyrosine kinase (*n* = 7), and others (*n* = 18; see Additional file 2, Table S2). Among the 48 drugs, 16 were screened in all PDCs and 32 were screened in 38 PDCs.

To investigate the genomic characteristics, we identified somatic mutations and CNVs using target-seq (*n* = 98) and classified four molecular subtypes (C1–C4) from gene expression profiles (*n* = 102; Additional file 2, Table S1). Next, we checked whether the PDCs effectively recapitulate the mutations and expression profiles of the solid tumors. Owing to the dominance of LUAD in our cohort (> 80%), we compared PDC gene expression profiles with TCGA-LUAD dataset (*n* = 230). As expected, the PDC gene expression profile resembled with tumor samples and was separated from normal adjacent tissues (Fig. 1B) [10]. Constitutive somatic gene mutations were similar in PDCs and TCGA samples. The recurrence of *TP53*, *RB1*, and *BRAF* mutations was highly preserved in both PDC and TCGA samples. The *EGFR* mutation frequency was higher in PDCs, whereas the recurrence of *KRAS*, *KEAP1*, and *STK11* mutations was lower than that of TCGA samples not shown in Fig. 1B. Thus, somatic mutations in *TP53* (47%), *EGFR* (29%), and *RB1* (8%) were frequently observed in PDC models (Fig. 1C). Moreover, *MET* (10%), *CDK4* (6%), and *MDM2* (6%) variants, as well as *EML4*-*ALK* (4%) and *CD74*-*ROS* (2%) fusion genes, were detected.

Before in-depth analysis, the most frequent *TP53* and *EGFR* mutations were additionally categorized according to selectivity or functionality [22, 47]. *TP53* mutations were divided into hotspot (known as gain-of-function; 5.9%; R175, G245, R248, R249, R273, R282) and non-hotspot (unknown or loss-of-function; 41.2%) mutations. *EGFR* mutations were categorized into (1) TKI-targetable single variant (TARGET: L858R and exon 19 deletion = 19.6%), (2) T790M acquisition (T790M^aq^: TARGET and T790M = 2.9%), (3) non-other-specified (NOS) single variant except TARGET (4.9%), and (4) NOS acquisition (NOS^aq^; TARGET and NOS = 2%; Table 1) [22, 47].

**Table 1:** Clinical characteristics of four RNA-subtypes

	RNA subtype	C1 (*n* = 13, 12.7%) Inflammatory	C2 (*n* = 45, 44.1%) EMT	C3 (*n* = 19, 18.6%) Stemness	C4 (*n* = 25, 24.5%) EGFR-dominant	*P*^a^
Overall Survival ^b^	HR (95% CI)	0.68 (0.34-1.36)	0.92 (0.60-1.42)	6.77 (3.73-12.27)	0.59 (0.35-0.97)	< 0.001
	OS (month, median)	22.8	23.0	4.2	36.7	
Progression-free Survival ^b^	HR (95% CI)	0.79 (0.37-1.66)	1.41 (0.87-2.28)	2.76 (1.35-5.61)	0.53 (0.31-0.91)	0.020
	PFS (month, median)	3.90	2.20	2.15	5.50	
Smoking	Pack (year, median)	9.00	0.20	29.00	0.00	0.176
Sex	Male (*n* = 57, 55.9%)	7 (6.9%)	23 (22.6%)	17 (16.7%)	10 (9.8%)	0.009
	Female (*n* = 45, 44.1%)	6 (5.9%)	22 (21.6%)	2 (2.0%)	15 (14.7%)	
Cancer Type	SCLC (*n* = 6)	0 (0.0%)	2 (2.0%)	4 (3.9%)	0 (0.0%)	0.015
	NSCLC (*n* = 96)	13 (12.8%)	43 (42.2%)	15 (14.7%)	25 (24.5%)	
Sample timing	Baseline (*n* = 31)	5 (4.9%)	13 (12.7%)	9 (8.8%)	4 (3.9%)	0.135
	Post (*n* = 71)	8 (7.8%)	32 (31.4%)	10 (9.8%)	21 (20.6%)	
EGFR-TKI therapy group	Sample size (*n* = 27)	2 (7.4%)	15 (55.6%)	0 (0.0%)	10 (37.0%)	0.038
	BASELINE (*n* = 9)	1 (3.7%)	5 (18.5%)	0 (0.0%)	3 (11.1%)	
	POST1 (*n* = 7)	0 (0.0%)	3 (11.1%)	0 (0.0%)	4 (14.8%)	
	POST2 (*n* = 4)	0 (0.0%)	1 (3.7%)	0 (0.0%)	3 (11.1%)	
	POST3 (*n* = 7)	1 (3.7%)	6 (22.2%)	0 (0.0%)	0 (0.0%)	
Target-seq	Sample size (*n* = 98)	13 (13.3%)	43 (43.9%)	17 (17.3%)	25 (25.5)	
TP53-mutation	Non-hotspot (*n* = 42)	4 (4.1%)	9 (9.2%)	8 (8.2%)	21 (21%)	< 0.001
	Hotspot (*n* = 6)	1 (1.0%)	2 (2.0%)	3 (3.1%)	0 (0.0%)	
	Wild-type (*n* = 50)	8 (8.2%)	32 (33%)	6 (6.1%)	4 (4.1%)	
EGFR-mutation	Target (*n* = 20)	3 (3.1%)	6 (6.1%)	2 (2.0%)	9 (9.2%)	0.258
	NOS^aq^ (*n* = 2)	0 (0.0%)	2 (2.0%)	0 (0.0%)	0 (0.0%)	
	T790M^aq^ (*n* = 3)	0 (0.0%)	1 (1.0%)	0 (0.0%)	2 (2.0%)	
	NOS (*n* = 5)	0 (0.0%)	3 (3.1%)	2 (2.0%)	0 (0.0%)	
	Wild-type (*n* = 68)	10 (10.2%)	31 (31.6%)	13 (13.3%)	14 (14.3%)	

^a^*P*-values were acquired from chi-square (categorical variables), ANOVA (quantitative variables), and log-rank (survival variables) tests

^b^*HR* Hazard ratio, *CI* Confidence interval, *OS* Overall survival, *PFS* Progression-free survival

^c^ Responder is complete or partial response, and non-responder is stable or progressive disease

### Molecular subtype classification and targeted drug candidate identification

**Fig. 2:**
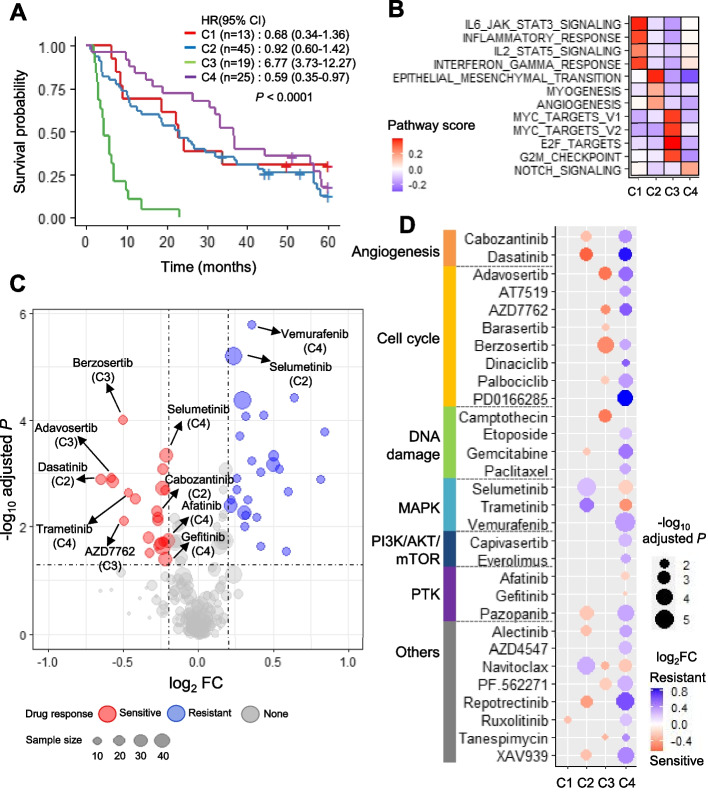
PDC molecular subtypes and their drug responses. **A** Overall survival curve plot of the four molecular subtypes. *P*-values were acquired using the log-rank test. Hazard ratio (HRs) 95% confidence intervals (CIs) were determined using the Cox model. **B** Heatmap indicating average scores of activated pathways for each subtype identified using the limma test (*P* < 0.001). **C** Volcano plot to test drug sensitivity of all possible pairs of subtypes and drugs. The x-axis is the log-scale fold change (log_2_ FC) to subtype’s average AUC divided by the rest samples’ average AUC. The y-axis is the *P*-value performed by Wilcoxon rank-sum test. Drug–subtype pairs are labeled. **D** Heatmap of drug–subtype pairs determined from response sensitivity tests for each subtype (*P* ≤ 0.05 and log_2_ FC ≤ 0.2). Only 30 drugs to pass *P*-value cutoff for each subtype were denoted. The size of each circle showed log-scale *P*-values, and circle was colorized by log_2_ FC

PDC samples could be classified into four molecular subtypes using gene expression profile by NMF clustering. To extrapolate the clinical characteristics for each subtype, we interrogated patient clinical profile encompassing histologic type, survival, smoking, and EGFR-TKI therapy record (Fig. 2 and Table 1). To uncover regulatory program for each molecular subtype, variant enrichment, and pathway regulation scores were assessed from multi-omics profile. In brief, subtype C1 was associated with a good outcome shown in Fig. 2A, and was activated in inflammatory and IL6-JAK-STAT3 signaling pathways; subtype C2 was associated with a modest outcome, dominance of *TP53*/*EGFR* wild-type, upregulation of epithelial-to-mesenchymal transition (EMT), and enrichment of osimertinib-resistant group (POST3) PDCs; subtype C3 was associated with the worst outcome, long-term smoking males, SCLC, *TP53* hotspot mutation, *MYC* activation, and fasten G2M checkpoint; by contrast, subtype C4 exhibited the best survival, frequent *TP53* non-hotspot mutation, the dominance of EGFR-TKI TARGET mutation, and NOTCH signaling activation (Fig. 2A, B). We also demonstrated the survival significance corresponding to C1–C4 subtype gene sets using additional transcriptome datasets (*n* = 1587; see Fig. S1A-B in Additional file 3 and Table S3 in Additional file 2). Upregulation of the C3 gene set concurrently exhibited the worst outcomes (*P* < 0.001 and hazard ratio [HR] = 2.6). When additionally demonstrating from known up-regulated genes of embryonic stem cell, we could observe the activation of human embryonic stem cell genes, and target genes’ upregulation of transcription factor *MYC* and *SOX2* (Fig. S2 in Additional file 3). The activation of C1 and C2 subtype genes significantly showed better prognosis (*P* < 0.05 and HR < 0.79). Our subtype classification sustained a global prognostic signature for lung cancer. Finally, we could summarize the subtypes based on the following regulatory pathways: C1, inflammatory; C2, EMT-like; C3, stemness; and C4, *EGFR*-dominant.

Sensitive drug candidates exhibited remarkable concordance with the previously identified regulatory pathways for each molecular subtype (*P* < 0.05 and |log_2_ fold change (FC)| < 0.2; FC was assessed to compare average drug AUC values between corresponding subtype and another group; Fig. 2C). The C1 inflammatory subtype was sensitive to only ruxolitinib (JAK1/2) that targets the JAT/STAT pathway (Fig. 2B-D). The C2 EMT-like subtype showed MAPK class drug resistance and sensitivity to dasatinib (angiogenesis and SRC inhibitor), cabozantinib (VEGFA inhibitor), miscellaneous class repotrectinib (ROS inhibitor), and XAV939 (WNT-TNKS-β-catenin inhibitor). The C3 stemness subtype was sensitive to the top five-ranked cell cycle inhibitors. The C4 *EGFR*-dominant subtype exhibited the strongest resistance to most drug classes except MAPK inhibitors (selumetinib and trametinib) and EGFR-TKIs (gefitinib and afatinib). Notably, targets of predicted drugs belonged to pathways that were found to be activated in each subtype (Fig. 2B).

### Predicting drugs for variants and dissecting the molecular subtype of EGFR-mutated PDCs

**Fig. 3:**
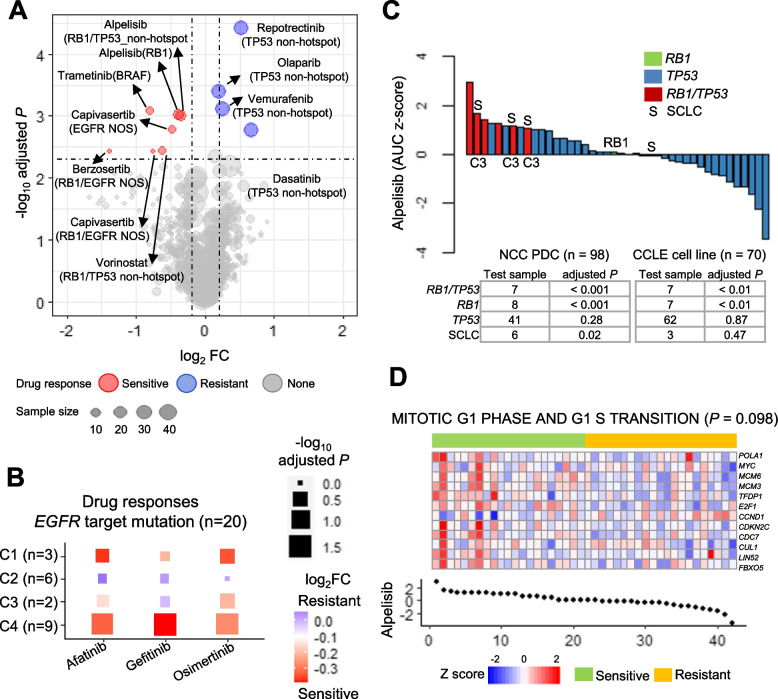
Drug candidates according to mutated cases. **A** Volcano plot to test drug sensitivity for single or double mutations (*P* ≤ 0.005 and log_2_ FC ≤ 0.2). **B** Heatmap to summarize the three EGFR-TKIs’ sensitivity of *EGFR*-target (L858R and exon 19 deletion; n = 20) mutated PDCs classified by four molecular subtypes. *P*-values (rectangle size) were acquired by Wilcoxon rank-sum test, and fold changes (FCs; color-coded) were calculated to compare target mutation group with the wild-type. The number of mutation cases for each subtype is denoted on the y-axis. **C** Waterfall plot of the alpelisib response. *RB1*, *TP53*, and *RB1*/*TP53* mutated PDCs are denoted. SCLC (S) and molecular subtype are labeled. The drug response comparison between mutation and wild-type investigated in both PDCs and cell lines, summarized in a table. **D** Heatmap of the alpelisib gene signature (*n* = 12) extracted from machine-learning and GSEA (pathway “MITOTIC G1 PHASE AND G1 S TRANSITION”, *P* < 0.1). PDCs were divided into drug-sensitive and -resistant groups, and the normalized AUC values are presented as a dot plot

To interrogate drug candidates for variants, we tested the difference in drug responses of single and co-occurrent mutation cases (*P* < 0.005 and |log_2_ FC| < 0.2; Fig. 3A). Co-mutated cases were also explored using the Fisher’s exact test (*P* < 0.25; see Additional file 3, Fig. S3). Unexpectedly, *EGFR*-TARGET mutated PDCs showed a relatively modest response to three EGFR-TKIs (afatinib *P* = 0.17, gefitinib *P* = 0.19, osimertinib *P* = 0.08). To uncover *EGFR*-mutated cells’ molecular features interfering with the EGFR-TKI response, we dissected TKI TARGET mutations according to our four molecular subtypes (Fig. 3B). The C4 *EGFR*-dominant subtype was the most sensitive to all EGFR-TKIs, and the C1 inflammatory subtype also showed an especially good response to afatinib and osimertinib. Mutated cases (*n* = 2) of the C3 stemness subtype was insufficient for statistical test. Finally, mutated cases (*n* = 6) of the C2 EMT-like subtype did not respond to any EGFR-TKIs. Additionally, both T790M^aq^ and NOS^aq^ (*EGFR* R776G and I744M with L858R) PDCs were TKI-sensitive. In the *EGFR* NOS type mutations, G719A was observed, and it comprised 11.5% among the NOS mutations in previous NSCLC study, and patient-derived xenografts demonstrated that the mutation was resistant to osimertinib [48]. The remaining NOS mutations excluded exon 18–21 and had a low possibility of finding a structure-based therapeutic target [48]. Therefore, we concluded that the remaining NOS group mutations were not targetable by EGFR-TKIs. Especially, *EGFR* TARGET mutated PDCs classified to EMT-like subtype exhibited low response to EGFR-TKIs. Thus, our molecular subtype of *EGFR* mutations revealed that these PDC models can be used to verify the heterogeneous tumor environment affecting drug responses.

When interrogating drug candidates for mutations, *BRAF* variants were sensitive to trametinib (Fig. 2A). The *TP53* non-hotspot exhibited resistance to olaparib (PARP inhibitor), repotrectinib, and dasatinib. *RB1* variants responded well to alpelisib. Although *TP53* mutation was not associated with any drug response (*P* < 0.28), *RB1*/*TP53* co-mutated samples exhibited certain sensitivity that was stronger than *RB1* (Fig. 3A). *RB1*/*TP53* mutations were mostly enriched in SCLC (*n* = 4; *P* < 0.001) but were also observed in NSCLC (*n* = 3; Additional file 3, Fig. S3). We additionally investigated alpelisib sensitivity comparing PDCs with cell lines (*n* = 70; SCLC n = 7). Notably, although alpelisib inhibited SCLC (Fig. 3C), *RB1*/*TP53* variants (*P* < 0.001) were more strongly inhibited than SCLC (*P* = 0.02). However, in contrast to *RB1/TP53* (*P* < 0.01), we failed to identify SCLC sensitivity to alpelisib (*P* = 0.47; Fig. 3C) using PDCs. Meanwhile, another PIK3-AKT target drug capivasertib (AKT1) was sensitive in *RB1*/*EGFR*-NOS and *EGFR*-NOS cases. When dissecting details, two of the five (*RB1*/*EGFR*-NOS) PDCs was classified to SCLC type additionally harboring *TP53* mutations (Fig. S3B in Additional file 3) [48]. Therefore, we could infer that *RB1*/*EGFR*-NOS PDC cases accompanied the dependency to SCLC type. Therefore, PI3K-AKT class drugs detected from SCLC or *RB1*/*TP53* cells also affect *EGFR*-NOS mutations. Collectively, mutation-based drug detection result emphasize that the genomic characteristics could predict both the cancer drug response and histological lung cancer type SCLC (Additional file 2, Table S1). Furthermore, our PDC model predicted alpelisib as an appropriate treatment for SCLCs, which was not identified in cell lines (Fig. 3C).

To delineate the drug susceptibility of alpelisib from transcriptome, we extracted the transcriptome signature for each drug using machine-learning approaches. The response to alpelisib was modulated by a transcriptome signature enriched “MITOTIC G1 PHASE AND G1 S TRANSITION” pathway gene set (Fig. 3D, and Additional file 2, Table S4). Upregulation of *MYC*, *E2F1*, and *CCNB1* expression led to resistance to alpelisib. Previously, we uncovered that stem-like C3 subtype exhibited both *MYC* up-regulation, and SCLC enrichment (Additional file 3, Fig. S2). *MYC* facilitates SCLC tumorigenesis [49]. Moreover, our SCLC PDCs predicted the response to alpelisib better than cell lines (Fig. 3C). Therefore, transcriptome gene signature extracted by machine-learning determined alpelisib response for SCLC like *RB1*/*TP53*.

### FOXM1 over-expression in SCLC type and sensitivity to cell cycle inhibitors

**Fig. 4:**
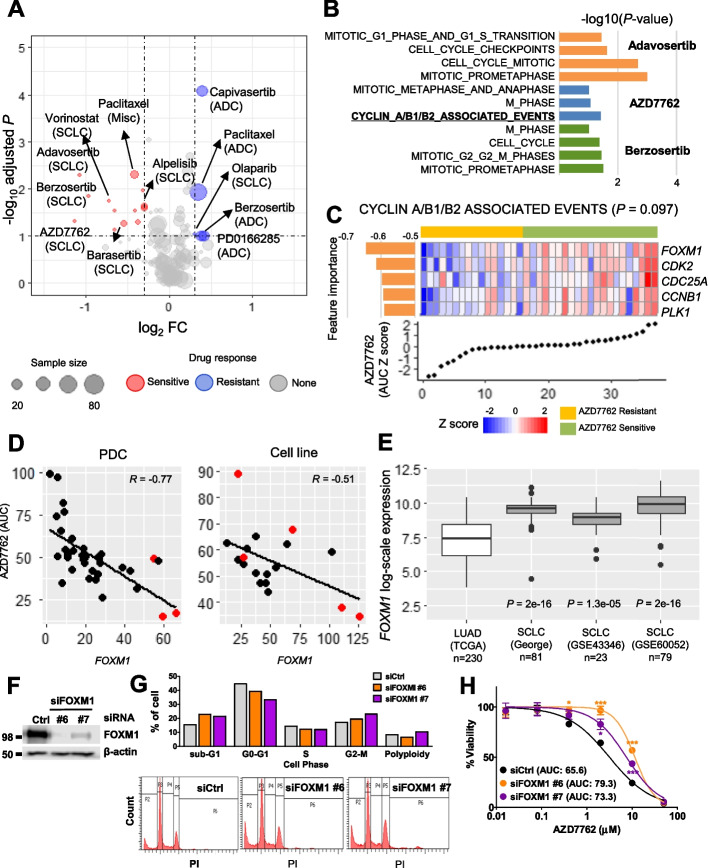
Sensitivity of lung cancer types to drug candidates. **A** Volcano plot for each lung cancer type. **B***P*-value bar plot of gene set enrichment analysis of transcriptome signatures for three drugs acquired from machine learning. **C** Five genes were associated with the cell cycle pathway. PDC gene expression heatmap for five genes detected in machine-learning feature importance and “CYCLIN A/B1/B2 ASSOCIATED EVENTS” pathway, and normalized AZD7762 AUCs. **D** Scatter plots for AZD7762 AUC and *FOXM1* expression for PDCs and cell lines. **E***FOXM1* expression comparison among four lung cancer cohorts between SCLC and LUAD. *P*-values were determined using the Wilcoxon rank-sum test between SCLC and LUAD. **F** Protein levels of FOXM1 and β-actin in siRNA-transfected H69 cells analyzed using western blotting. Ctrl represents the control, and siFOXM1-#6 and siFOXM1-#7 are *FOXM1* siRNAs. **G** Cell cycle phase assessment according to control and *FOXM1* siRNA conditions of H69 cells. After incubation, the cells were analyzed using flow cytometry to evaluate the DNA content. Representative DNA content profiles from three independent experiments are shown. Bar plots present the proportions of cells in cell cycle phases (upper panel) and cell counts (y-axis) based on propidium iodide (PI) staining (x-axis; lower panel). **H** Drug response curves for testing AZD7762 sensitivity in control siRNA (Ctrl) and siRNA #6- and #7-transfected H69 cells. The half-maximal inhibitory concentration (IC_50_) of AZD7762 siRNA-transfected cells is shown in the panel. All experiments were performed in quadruplicate. The values represent the mean ± SEM (Student’s *t*-test, *P < 0.05; ***P < 0.001)

For further in-depth exploration of the candidate drugs for treating different lung cancer types, we investigated the drug responses of ADC, SCLC, and miscellaneous cancers. SCLC was sensitive to drugs targeting cell cycle pathways (adavosertib, barasertib, berzosertib, and AZD7762) and DNA damage (vorinostat; *P* < 0.1 and |log_2_FC| < 0.3; Fig. 4A). The SCLC sensitivity to alpelisib was relatively lower than that to cell cycle drugs according to the FC value. The machine-learning signatures of three drugs (adavosertib, AZD7762, and berzosertib) consistently contained cell cycle pathways (Fig. 4B, and Additional file 2, Table S4). The transcriptome profile revealed the activation of cell cycle genes in SCLC. Moreover, the DNA damage response and RAS signal were downregulated in NSCLC (Additional file 3, Fig. S4). Collectively, these comprehensive pharmacogenomics result suggest the role of cell cycle inhibition in treating SCLC types.

PDCs and cell lines showing *FOXM1* upregulation exhibited AZD7762 sensitivity (Fig. 4C, D; PDC *R* = –0.77; cell line *R* = –0.51). *FOXM1* was clearly over-expressed in SCLC compared to that in LUAD (Fig. 4E) [10, 50–52]. To evaluate whether *FOXM1* is a plausible target to AZD7762 for SCLC, we transfected H69 cells with small interfering RNAs (siRNAs) against *FOXM1* (#6 and #7, siFOXM1) and control siRNA and measured the FOXM1 protein level using western blotting. The FOXM1 expression level was decreased in siFOXM1 (Fig. 4F). siFOXM1 cells also exhibited decreased cell proliferation compared to siControl-transfected cells. However, G2-M phase arrest was detected in siFOXM1 H69 cells (Fig. 4G). siFOXM1 became more resistant to AZD7762 (AUC FC > 1.1) than siControl-transfected cells (Fig. 4H). Another cell lines H209 also presented the same result (see Additional file 3, Fig. S5). Therefore, we concluded that *FOXM1* plays essential role to SCLC participating in the G2-M phase, and can be targeted by cell cycle inhibitors.

### Pharmacogenomic analysis according to EGFR-TKI treatment status

**Fig. 5:**
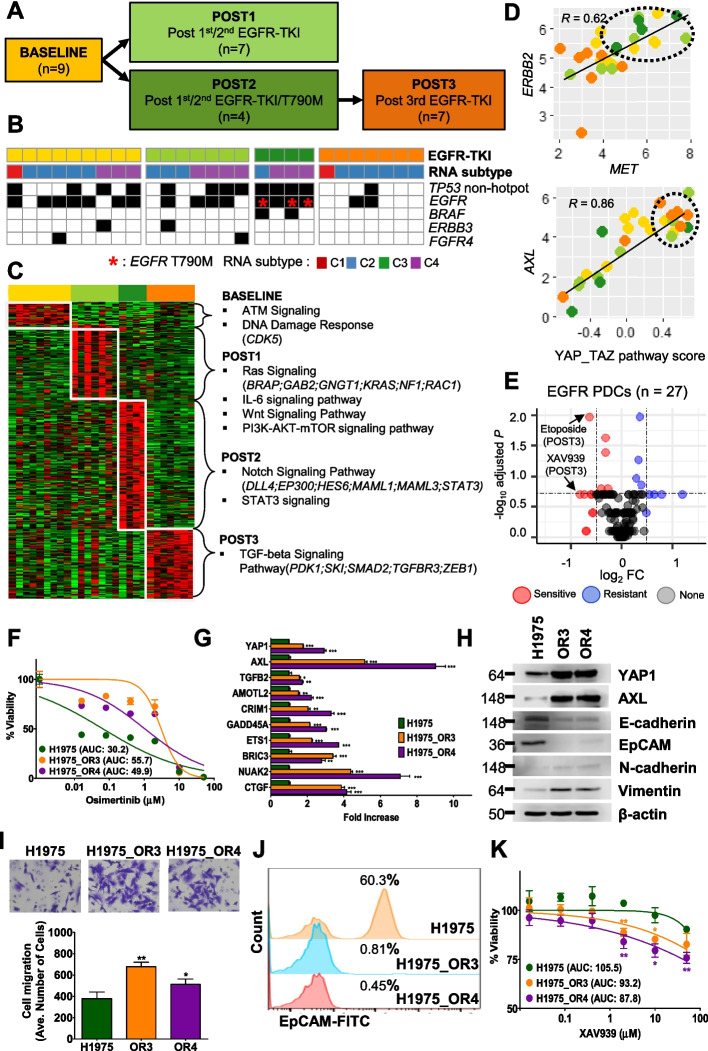
Genomic profile and drug sensitivity of PDCs, and functional validation using cell lines. **A** Therapeutic groups were categorized into two arms. The first arm was BASELINE (yellow) to POST1 (light green). The second was BASELINE to POST2 (dark green) to POST3 (orange). **B** Heatmap presenting the mutation profile, RNA subtype, and tumor mutation burden (TMB) of each group. In the POST1 group, the red asterisk indicates the *EGFR* double mutation including T790M. **C** Heatmap of the DEGs for the four groups. DEGs and pathways (GSEA *P* < 0.1) for each group are denoted on the right. **D** Two scatter plots of scores extracted from EGFR-TKI resistance pathway gene pairs. *ERBB2* and *MET* were upregulated in the POST2 group (dotted circle), and *YAP/TAZ*-*AXL* activated in the POST3 group (dotted circle). *R* indicates Pearson’s correlation coefficient, and the *P* value was acquired using the Wilcoxon rank-sum test from the AUC values. **E** Volcano plot to test drug sensitivity for each group. The x-axis is log_2_ fold change (FC) and the y-axis is *P*-value. **F** Drug response curves based on the viability of H1975, H1975_OR3, and OR4 cells treated with osimertinib. **G** Bar plots of *YAP1* and its target gene expressions. Fold increases (x-axis) in mRNA expression in each gene (y-axis) were assessed using RT-PCR in H1975, H1975_OR3, and OR4 cells. **H** The protein level expression assessment in three cells using western blot analysis. **I** Cell migration across the transmembrane. Cell morphology images are shown from three cases. The bar plot indicates the average number of migrating cells (y-axis) for each condition (x-axis). **J** EpCAM expression (x-axis) determined using flow cytometry. Graphs were analyzed using the FlowJo program. **K** Drug response curves treated with XAV939. The AUC of each condition for XAV939 is shown in the panel. All experiments were performed in quadruplicate. The values represent the mean ± SEM (Student’s *t-*test, *P < 0.05; **P < 0.01; ***P < 0.001)

Among our PDCs, we identified 27 EGFR-TKI-treated cases with corresponding NGS results (independently obtained in the clinic using tissue samples). As described in methods, we these patients into four groups (Fig. 5A). Primarily, POST3 samples (86%) were enriched in the C2 EMT-like subtype (Table 1). *EGFR* mutation-calling failure was observed in the clinical tumor biopsy NGS record of some patients from which BASELINE (*n* = 2) and POST2 (*n* = 1) PDCs were derived. The mutation-calling failure likely originated from differences in the platform between the clinical biopsy and laboratory PDC testing. Moreover, *TP53* non-hotspot mutations were present in all of the POST2 PDCs and were markedly absent in POST3 PDCs. *EGFR* T790M^aq^ was identified in only POST2 PDCs (Fig. 5B red asterisk); interestingly, *BRAF* mutations highly co-occurred with *EGFR* T790M^aq^ mutations (Fig. 5B and Additional file 3, Fig. S3). Thus, our PDC models significantly reflected *EGFR* mutation, acquisition, and extinction status according to EGFR-TKI therapies.

The four therapeutic groups exhibited distinct regulatory programs. To identify the resistance pathway, we applied two different methods. First, activated pathways were inferred from upregulated DEGs for each group (see Additional file 2, Table S5). The activation of ATM signaling and the DNA damage response according to *CDK5* upregulation were enriched in the BASELINE PDCs. POST1 PDCs predisposed to RAS, IL-6, WNT, and PI3K-AKT-mTOR signals were enriched with upregulated genes (*BRAP*, *KRAS*, and *NF1*). POST2 PDCs were enriched in NOTCH and STAT3 signaling, including *DLL4*, *EP300*, and *STAT3* over-expression. Finally, POST3 PDCs exhibited activated TGF-β signaling regulated by *PDK1*, *SMAD2*, *TGFBR3*, and *ZEB1*. The over-expression of TGF-β signaling and *ZEB1* demonstrated that osimertinib-resistant cancer exhibited EMT pathway activation (Fig. 5C).

We next assessed the activities of 12 knowledge-based therapeutic resistance pathways for each sample according to the four EGFR-TKI therapeutic groups (Additional file 2, Table S6). The *SERPINE1* signature was only activated in the BASELINE PDCs (Additional file 3, Fig. S6). The WNT, ERBB2, and MET pathway scores were increased in POST2 PDCs but were decreased in POST3 PDCs. The YAP/TAZ and PI3K-AKT pathways were elevated in POST3 PDCs. *MET* and *ERBB2* gene expression also showed positive correlations with activation in the POST2 PDCs (*R* = 0.62), whereas the POST3 PDCs exhibited inactivation in both pathways (Fig. 5D). The EMT-like POST3 PDCs exhibited the over-expression of both YAP/TAZ and *AXL* (*R* = 0.86; Fig. 5D).

Among the four EGFR-TKI groups, we explored the sensitivity of POST3 PDCs to etoposide and XAV939 (Fig. 5E). To predict drugs for 27 PDCs, we prepared an additional drug screening dataset by including the extended PDC set without available sequencing data (*n* = 70; Additional file 3, Fig. S7A). To make up for the lack of genomic profiles, we collected EGFR-TKI therapy and clinical NGS test medical records. We could assign these samples to the four groups (BASELINE, *n* = 12; POST1, *n* = 23; POST2, *n* = 17; POST3, *n* = 18). The extended PDC dataset showed remarkable concordance in patient categorization obtained using NGS results and medical records. Finally, POST3 PDCs (PDC *P* < 0.2, extended PDC *P* < 0.083; Fig. 5E) were sensitive to etoposide and XAV939. We found that POST3 PDCs exhibited higher sensitivity to XAV939 than etoposide. The machine-learning XAV939 gene signature revealed that the response to XAV939 also involved EMT-like molecular features (i.e., collagen formation and extracellular matrix organization; Additional file 2, Table S4).

To evaluate the previous finding about XAV939 selectivity and pathways, we created two cell lines—*EGFR*-T790M^aq^ (H1975) cells using POST2 PDCs and XAV939-resistant (H1975_OR3, H1975_OR4) cells using POST3 PDCs—with osimertinib exposure. Both cell lines acquired osimertinib resistance (FC > 1.7; Fig. 5F). TCGA pan-cancer and reverse transcription-quantitative polymerase chain reaction analyses (RT-PCR) revealed that osimertinib-resistant cells concurrently exhibited over-expression of YAP/TAZ target genes [46]. Ten target genes, including *YAP1* and *AXL,* were considerably upregulated in the osimertinib-resistant cell lines compared to that in parent H1975 cells (Fig. 5G). In particular, *AXL* was elevated with the highest FC (5.1–9.0), and YAP1 and AXL proteins showed consistent over-expression in osimertinib-resistant cell lines (Fig. 5H).

Next, we identified osimertinib resistance-related molecular subtypes and cellular alterations in POST3 PDCs. Since POST3 PDCs were classified as an EMT-like subtype (Fig. 5C), we assessed the EMT features of osimertinib-resistant PDCs. Two osimertinib-resistant PDCs showed decreased E-cadherin and EpCAM (epithelial markers) and increased N-cadherin and Vimentin (mesenchymal markers; Fig. 5H) expression levels. Subsequently, using flow cytometry, we established that cell surface EpCAM expression was considerably decreased in these cell lines (Fig. 5I). We further conducted a migration assay to verify these EMT-associated molecular changes (Fig. 5J). As expected, the two osimertinib-resistant PDCs had greater migration ability than H1975 cells. Collectively, these findings demonstrated that the two successfully generated osimertinib-resistant cell lines were representative of POST3 PDCs. Next, we checked if XAV939 had similar inhibitory effects on POST3 PDCs and H1975, H1975_OR3, and H1975_OR4 cells. XAV939 was not cytotoxic to H1975 cells (AUC = 105.5); however, H1975_OR3 (93.2) and OR4 (87.8) cells were clearly more sensitive to XAV939 (Fig. 5K). Overall, we validated that osimertinib resistance facilitates YAP/TAZ and AXL activation, and an EMT-like phenotype. Thus, XAV939 may be used to treat osimertinib-resistant tumors.

## Discussion

Our PDC platform was established with a lower labor burden and offers advantages of cost effectiveness and fast data acquisition compared with other platforms developed using PDXs, organoids, and cell lines. These advantages could increase the case number and, consequently, guaranteed the statistical significance and tumor heterogeneity of the test data. Our PDC platform currently includes 327 lung cancer cases and exhibits 77.7% success rates for an average 16.6 days duration time. Moreover, the PDC collection from 2020 achieved even better performance with an 83.9% success rate and 14.5 days duration time. Our platform performance exceeded that of a previous lung cancer screening [9]. These characteristics indicate that our platform is effective to operate in the medical field for patient-tailored precision medicine. Moreover, in EGFR-TKI therapeutic groups, we observed approximately 11% *EGFR*-mutation calling loss compared with the medical record and patient NGS profile. This is likely due to multiple factors contributing to differences among platforms, such as the biopsy type, tumor clonality, purity, and sequencing depth [53]. Meanwhile, our results were demonstrated using lung cancer cell lines. In further study, we have a plan to evaluate experimental demonstration from immortalized cells generated from PDCs. When adjusting the platform pipeline in the next collection, we expect to improve the accuracy of pharmacogenomic analysis.

Among molecular subtypes, stem-like type C3 exhibited the most significant up-regulation in SOX2 than other transcription factors. In previous reports, stemness-related transcription factors play a role to depend on cancer tissue type. SOX2 was required in early-stage ADC and SCLC, whereas OCT3, KLF4, and NANOG participate other cancer types [54]. Despite low frequency of SCLC, these were also enriched in this subtype. RB1/TP53 co-mutation was SCLC driver gene, and its depletion facilitate aberrant cell-cycle [15]. Additionally, *MYC* up-regulated in C3 type drives dynamic evolution of SCLC [55]. Therefore, our molecular subtype successfully classified these comprehensive regulatory features.

Through detailed EGFR-TKI response analysis, we showed that the PDC molecular subtype uncovers the clinical characteristics and resistance factors of lung cancer patients. Our subtype classification suggested therapeutic candidates according to regulatory program. In additional classification of EGFR TARGET mutations, we could uncover that molecular subtype implicated in EGFR-TKI responses of PDCs harboring EGFR mutations. In particular, the C2 subtype designated as EMT-like exhibited *FGFR2* over-expression. The *FGF7-FGFR2* over-expressing lung cancer-associated fibrosis (CAF) type robustly protects *EGFR*-mutated cancer cells to maintain osimertinib resistance [56]. CAF and *EGFR*-mutated co-cultured cells exhibit osimertinib resistance. Here, using our PDC models, we could directly assess EMT interference via EGFR-TKI resistance from spontaneous patient tumor clonal status. Moreover, our data showed that both the EMT-like subtype and osimertinib-resistant patients are sensitive to XAV939. Machine-learning gene signatures also revealed that regulatory programs that induce EMT-like characteristics were sensitive to XAV939.

The genomic variant *RB1*/*TP53* showed more sensitivity to alpelisib than SCLC, whereas SCLC exhibited sensitivity to cell cycle inhibitors. Interestingly, NSCLC to SCLC transformation co-occurred with EGFR-TKI resistance, and *RB1*/*TP53* loss-of-function mutation occurred earlier than expected in the cancer cell cycle [57]. Upregulation of *PIK3CA* mutation and PI3K/AKT pathway genes also occurred earlier during the transformation [58]. A patient-derived *EGFR*-mutant xenograft model verified that early PI3K/AKT pathway inhibition delays tumor growth in SCLC or NSCLC undergoing transformation. Importantly, our PDC model concurrently selected alpelisib, a PI3K/AKT inhibitor, for treating early-stage SCLC. Consequently, our results emphasize that progressive and de novo SCLC phenotypes are more sensitive to cell cycle inhibitors [15]. We suggest that strategic combinatorial therapy with anti-cancer drugs belonging to two different classes can block the progression of early-stage SCLC.

*EGFR*-mutated lung cancer patients responding well to TKIs eventually develop resistance. Particularly, osimertinib resistance develops via heterogeneous and complex mechanisms, making establishment of an effective therapeutic strategy difficult. Our therapeutic follow-up delineated resistance pathways activated by EGFR-TKI treatment [23]. *ERBB2* and *MET* were activated in POST1 PDCs. *BRAF* mutations significantly co-occurred with *EGFR*-T790M. Furthermore, YAP/TAZ was activated in POST4 PDCs. Our results suggest XAV939 for POST3 PDCs. Interestingly, BASELINE PDCs, similar to POST3 PDCs, responded to XAV939 (Additional file 3, Fig. S7B). Thus, first-line combinatorial therapy with EGFR-TKI and XAV939 seems plausible. Gefitinib and XAV939 acted synergistically in the combinatorial therapy of the *EGFR*-mutated H1975 cell line (combination index (CI) = 0.388; synergism CI < 0.9). We expect effective translation of these results into treatments for patients with osimertinib-resistant lung cancer.

Precision oncology in lung cancer is mainly based on gene-targeted chemotherapy; however, evasive mutations in target genes confound the prognosis. The PDCs developed in this study offer an advantage in tailoring patient-specific drugs and understanding the comprehensive molecular features of cancer. Importantly, our molecular subtypes reflected the PDC heterogeneity and recapitulated the drug response-mediated interference of EMT. However, as a limitation, we observed mutation-calling failure in a few EGFR-TKI treatment cases (11%), which was attributed multiple factors such as biopsy differences, tumor clonality, purity, and sequencing depth [53]. In the future, we expect to improve the accuracy of pharmacogenomic analysis by adjusting the platform pipeline.

## Conclusions

Our well-established pharmacogenomic platform effectively predicted drugs and response mechanisms for refractory lung cancer. Following this pilot study, we expect consistent use of the established PDC bank in unveiling comprehensive drug–target associations of clinical relevance.

## Supplementary Information

**Additional file 1.** Supplemental methods.

**Additional file 2: Table S1.** Clinical information and molecular subtypes of 102 lung cancer patients used to obtain PDCs. **Table S2.** The 48 drugs employed in the screening panel. We refer to the chemical or generic name of the drugs, and the target and class for each drug were classified. **Table S3.** Differentially expressed gene sets for the four molecular subtypes. **Table S4.** GSEA results (*P* < 0.1) using drug-associated gene signatures extracted with a machine-learning approach. **Table S5.** GSEA results (*P* < 0.1) acquired from analysis of the upregulated DEGs from four EGFR-TKI treatment groups. **Table S6.** EGFR-TKI resistance pathway and related gene sets.

**Additional file 3: Fig. S1.** Molecular subtype evaluation using six lung cancer cohorts (*n* = 1587). (A) Overall survival plots for each molecular subtype according to the subtype gene signature score. High and low groups were selected from the upper and lower quartiles. *P*-values and hazard ratios (HRs) were calculated using the log-rank test and Cox model. (B) HR forest plots for each subtype across six lung cancer cohorts. **Fig. S2.** Additional assessment of stemness scores for each molecular subtype. (A) A heatmap of average stemness scores according to molecular subtype. Stemness scores were assessed using GSVA from gene signatures: embryonic stem (ES) cell up-regulated genes (ES exp1, and ES exp2), and five transcription factors’ target genes as well as four PRC2 complex target signatures as control sets. (B) Boxplots of stemness scores according to subtypes. *P*-values were calculated by Wilcoxon rank-sum test to compare C3 and others. **Fig. S3.** Co-occurrent mutation case investigation. (A) Significant co-mutation pairs extracted using the Fisher’s exact test (recurrence > 5%). (B) The status of RB1/TP53 mutation and SCLC type. **Fig. S4.** Heatmap of DEGs between SCLC and NSCLC. The rows of the heatmap are the samples and the columns show the genes. Genes were extracted using limma (adjusted *P* < 0.005, |log2 FC| > 0.5) and the GSEA results (Table S3, Additional file 2) are summarized on the left. **Fig. S5.** Validation of the correlation of FOXM1 expression and AZD7762 sensitivity in H209 cells. (A) The protein levels of FOXM1 and β-actin in siRNA-transfected H209 cells (siFOXM1-#6, #7) analyzed using western blotting. Ctrl represents the control. H209 cells transfected with siRNAs were incubated for 48 h. (B) After incubation, the cells were analyzed using flow cytometry to evaluate the DNA content. Representative DNA content profiles from three independent experiments are shown. The graphs show the proportion of cells in each cell cycle phase. (C) Drug response curve of siRNA-transfected H209 cells treated with AZD7762 (x-axis). The area under the receiver operating characteristic curve (AUC) values of AZD7762 in siRNA-transfected cells are shown in the panel. All experiments were performed in quadruplicate. The values represent the mean ± SEM (Student’s t-test, *P < 0.05; ***P < 0.001). **Fig. S6.** Pathway score bar plots of six EGFR-TKI resistance signatures according to four EGFR-TKI therapeutic groups. The numbers on each bar plot indicate the *P* values obtained using the Wilcoxon rank-sum test. **Fig. S7.** Drug candidates extracted from EGFR-TKI group PDCs (*n* = 27) and extended EGFR PDCs (*n* = 70). (A) Volcano plots for EGFR-TKI groups of two datasets. The x-axis indicates the log2-fold change between drug responses and the y-axis shows the log-scale adjusted *P*-value. Red circles indicate sensitivity whereas blue circles indicate resistance. (B) Bar plots for etoposide and XAV939 AUC in both datasets. The x-axis indicates EGFR-TKI groups and the y-axis indicates AUC values. P values were obtained using the Wilcoxon rank-sum test.

## Data Availability

Next-generation sequencing files of refractory lung cancer PDC samples have been deposited in the National Center for Biotechnology Information Gene Expression Omnibus (GSE165611) database for RNA-seq and in the Sequence Read Archive (PRJNA694788) for target-seq.

## Abbreviations

CAF: Cancer-associated fibrosis
CI: Combination index
DEG: Differentially expressed gene
EGFR: Epidermal growth factor receptor
EMT: Epithelial-to-mesenchymal transition
FC: Fold change
HR: Hazard ratio
LUAD: Lung adenocarcinoma
NGS: Next-generation sequencing
PDC: Patient-derived tumor cells
SCLC: Small-cell lung cancer
siRNA: Small interfering RNA
TCGA: The Cancer Genome Atlas
TKI: Tyrosine kinase inhibitor
