# Supplementary material for: Patient-derived cell-based pharmacogenomic assessment to unveil underlying resistance mechanisms and novel therapeutics for advanced lung cancer
Source: J Exp Clin Cancer Res. 2023 Jan 30;42:37. doi: 10.1186/s13046-023-02606-3 (PMC9885631; doi:10.1186/s13046-023-02606-3)
Supplement: Supplementary file 1 — Additional file 1. Supplemental methods. [file 13046_2023_2606_MOESM1_ESM.docx]

**Supplementary methods**

***PDC establishment of advanced or refractory lung cancer***

To establish PDC, pleural effusions (92.2%), pericardial effusions (4.9%), ascites (2.0%) and tissues (1.0%) were obtained from lung cancer patients at the National Cancer Center (Additional file 1: Table S1). PDCs were isolated from liquid samples (pleural effusions) by density gradient centrifugation using Lymphocyte Separation Medium (LSM, #091692249; MP Biomedicals, Solon, OH, USA) following the manufacturer’s instructions. For tissue samples, tumor tissues were minced finely and washed with RPMI. After washing, cells from effusion or tissue samples were cultured in AR-5 medium (5% FBS, 1 X GlutaMAX (Thermo Fisher Scientific, Waltham, MA, USA), 1 X Insulin-Transferrin-Selenium (ITS, Thermo Fisher Scientific), 1% penicillin/streptomycin, 50 nM hydrocortisone, 1 mM sodium pyruvate and 1 ng/ml EGF in RPMI 1640) at 37°C in a 5% CO_2_ atmosphere. The media were changed carefully every 2~3 days until the cells were stabilized in the flask.

Human lung cancer cell lines were cultured for validation experiments. H1299 and H1975 cells were purchased from ATCC; A549, H69 and H209 cells were purchased from Korea Cell Bank, and PC9 cells were purchased from RIKEN. All cell lines were maintained in RPMI 1640 with 10% FBS.

***Isolation of genomic DNA and RNA for next-generation sequencing***

Genomic DNA was extracted from PDC using the AllPrep DNA/RNA Mini kit and handled by a QIAcube automatic instrument (Qiagen, Germantown, MD, USA) followed by hgDNA 3.1 cultured cell protocols in a MagNA Pure 24 System (Roche, Switzerland). DNA from each sample was fragmented by acoustic shearing on a Covaris S2 instrument and then hybridized with RNA probes, SureSelect XT Custom Kit library. The high-quality libraries were pooled and sequenced on the Illumina NovaSeq 6000 platform (Illumina) with 150 bp paired-end by following the manufacturer’s protocols. Image analysis was performed using NovaSeq6000 control Software version 1.3.1, and the output base calling data were demultiplexed with bcl2fastq version v2.20.0.422, generating fastQC files.

Total RNA was extracted from PDC using the RNeasy® Mini kit and AllPrep DNA/RNA Mini kit and handled by a QIAcube automatic instrument (Qiagen). RNA libraries were prepared as paired-end reads with a length of 100 bases using an Illumina TrueSeq Stranded mRNA Sample Preparation kit according to the manufacturer’s protocols. The libraries were sequenced as paired-end reads (2 x 150 bp) using the Illumina NovaSeq 6000.

***Evaluation of cell cycle inhibitors effective for SCLC and the associated gene FOXM1***

We investigated the transcriptome characteristics of SCLC from differentially expressed gene (DEG) analysis to compare SCLC with NSCLC using limma [1]. Next, gene set enrichment analysis (GSEA) was performed using the upregulated DEGs for each group by referring to WikiPathways [2,3] To evaluate drugs and genes for SCLC, we additionally investigated drug sensitivity data for SCLC versus NSCLC from cell lines. Our drug response screening result for AZD7762 was acquired from 13 NSCLC cell lines and 5 SCLC cell lines, similar to our PDCs. To assess the similarity of drug signature genes between cell line and PDC, We additionally assessed Pearson correlation coefficient [4].

The siRNAs targeting FOXM1 (Hs_FOXM1_6 and Hs_FOXM1_7) and the control siRNA were purchased from QIAGEN (Foster City, CA, USA). H69 and H209 cells were transiently transfected with siRNAs using a NEPA21 electroporator (NEPA GENE, Chiba, Japan). Suspension cells (1 x 10^6^) with 100 pmole siRNA in 100 μl OPTI MEM media per cuvette were subjected to electroporation with program No. 5 following the manufacturer’s instructions.

***Generation of osimertinib-resistant cell lines***

H1975 cells on 100-mm dishes were exposed to 50 nM osimertinib during incubation. After 3 weeks, the cells were cultured in osimertinib-free media for 1 week to expand resistant clones. After 4 osimertinib treatment cycles (3 weeks in 50 nM osimertinib - 1 week in free), the cells were exposed to 500 nM osimertinib for 3 weeks and then cultured in osimertinib-free medium for 1 week to obtain resistant clones. Osimertinib-resistant H1975 (H1975_OR3 and OR4) cells were subjected to an osimertinib treatment cycle (3 weeks in 500 nM osimertinib - 1 week osimertinib free).

***Immunoblot analysis and antibodies***

Cell lysates were prepared for immunoblotting, which was performed as described previously [5]. The developed proteins were visualized using a C-Digit Blot Scanner (LI-COR Bioscience, Lincoln, NE, USA). Primary antibodies targeting the following proteins were used: FOXM1 (sc-271746, Santa Cruz Biotechnology, Santa Cruz, CA, USA), β-actin (sc-47778, Santa Cruz Biotechnology), YAP1 (#14074, CST), AXL (#8661, CST), E-cadherin (ab1416, Abcam, Cambridge, UK), EpCAM (ab71916, Abcam), N-cadherin (#13116, CST), and vimentin (#5741, CST).

***Quantitative reverse transcription PCR (RT–qPCR)***

Total RNA was extracted from H1975 and H1975_OR cells using TRIzol reagent (Invitrogen, Carlsbad, CA, USA) and then used for cDNA synthesis using the SuperScript^TM^ III First-Strand kit (Invitrogen). RT–qPCR assays were performed using SYBR Green I Master Mix (04707516001; Roche, Manheim, Germany) and a real-time PCR system (05815916001; Roche). Glyceraldehyde 3-phosphate dehydrogenase (GAPDH) mRNA was used as a normalization control.

***Cell migration assay***

Cell migration was evaluated by using 24-well plates containing cell culture inserts with 8-μm pore PET track-etched membrane Transwell chambers (353097; Corning, Corning, NY, USA). H1975 and H1975_OR cells were seeded at 1 x 10^5^ cells/well in 100 μl of 1% FBS RPMI culture medium in the upper chamber of permeable Transwell supports. The lower chamber was filled with 500 μl of RPMI supplemented with 10% FBS, and cell migration was tracked for 24 hrs. Migrating cells were stained with 0.1% crystal violet and then counted in three randomly selected fields per well under a light microscope.

***DNA ploidy and FACS analysis***

Cells were subjected to DNA ploidy and FACS analysis as described previously [5]. H1975 and H1975-OR cells (1 x 10^6^) were incubated with 2 μl of anti-EpCAM antibody conjugated with FITC (130-113-263; Miltenyi Biotec, Gladbach, Germany) for 10 min at 4°C in the dark. After incubation, the cells were washed with PBS and analyzed using a FACSCaliber (BD).

***Reference***

1. Ritchie ME, Phipson B, Wu D, Hu Y, Law CW, Shi W, et al. Limma powers differential expression analyses for RNA-sequencing and microarray studies. Nucleic Acids Res. 2015;43:e47.

2. Wang J, Vasaikar S, Shi Z, Greer M, Zhang B. WebGestalt 2017: A more comprehensive, powerful, flexible and interactive gene set enrichment analysis toolkit. Nucleic Acids Research. 2017;45:W130–7.

3. Martens M, Ammar A, Riutta A, Waagmeester A, Slenter DN, Hanspers K, et al. WikiPathways: Connecting communities. Nucleic Acids Res. 2021;49:D613–21.

4. Ghandi M, Huang FW, Jané-Valbuena J, Kryukov G V., Lo CC, McDonald ER, et al. Next-generation characterization of the Cancer Cell Line Encyclopedia. Nature. 2019;569:503–8.

5. Jun DW, Hwang M, Kim YH, Kim KT, Kim S, Lee CH. DDRI-9: a novel DNA damage response inhibitor that blocks mitotic progression. Oncotarget. 2016;7:17699–710.
