# Supplementary material for: Patient-derived cell-based pharmacogenomic assessment to unveil underlying resistance mechanisms and novel therapeutics for advanced lung cancer
Source: J Exp Clin Cancer Res. 2023 Jan 30;42:37. doi: 10.1186/s13046-023-02606-3 (PMC9885631; doi:10.1186/s13046-023-02606-3)

**Figure S1.** Molecular subtype evaluation using six lung cancer cohorts (n=1587). A) Overall survival plots for each molecular subtype according to subtype gene signature score. High and low group were selected from upper and lower quartile. P-values and hazard ratio (HR) were calculated using the log-rank test and Cox model. B) HR forest plots for each subtype across six lung cancer cohorts.


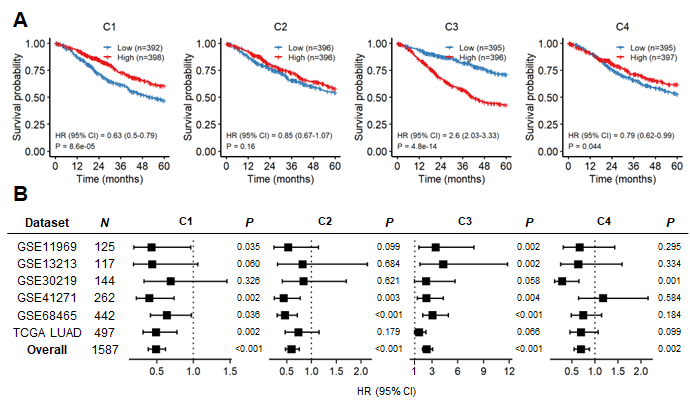


**Figure S2.** Additional assessment of stemness scores for each molecular subtype. (A) A heatmap of average stemness scores for each subtype assessed using GSVA from gene signatures: embryonic stem (ES) cell gene expression (ES exp1, and ES exp2), and five transcription factors’ target genes as well as four PRC2 complex target signatures. (B) Boxplots of stemness scores for each subtype. *P*-values were calculated by Wilcoxon rank-sum test to compare C3 and others.


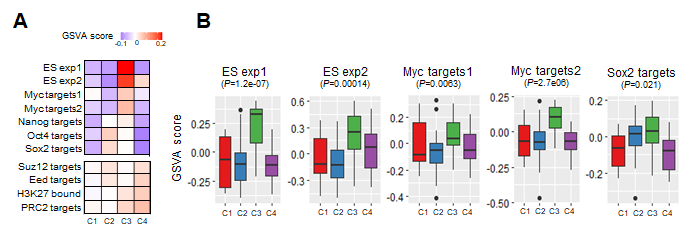


**Figure S3.** Co-occurrent mutation case investigation. A) Significant co-mutation pairs extracted using the Fisher’s exact test (recurrence>5%). B) The status of *RB1*/*TP53* mutation and SCLC type.


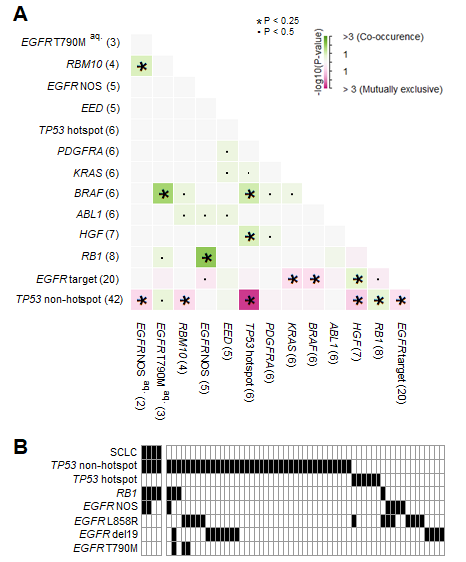


**Figure S4.** Heatmap of DEGs between SCLC and NSCLC. The rows of the heatmap are the samples and the columns show the genes. Genes were extracted using limma (adjusted P < 0.005, |log2 FC| > 0.5) and the GSEA results (Table S3, Additional File 2) are summarized on the left.


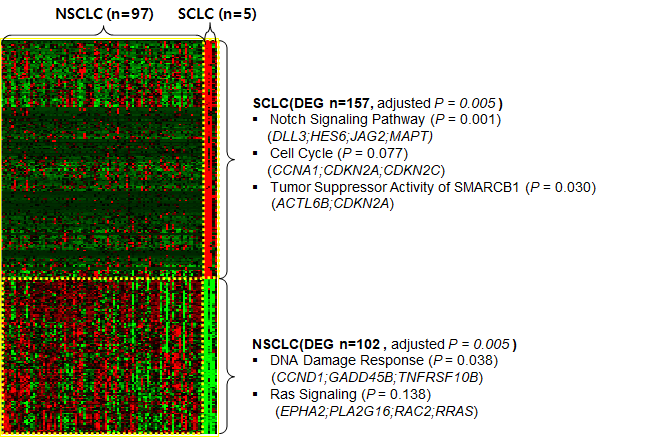


**Figure S5.** Validation of the correlation of *FOXM1* expression and AZD7762 sensitivity in H209 cells. A) The protein levels of FOXM1 and β-actin in siRNA-transfected H209 cells (siFOXM1-#6, #7) were analyzed using western blotting. Ctrl represents the control. H209 cells transfected with siRNAs were incubated for 48 h. B) After incubation, the cells were analyzed using flow cytometry to evaluate DNA contents. Representative DNA content profiles from three independent experiments are shown. The graphs show the proportion of cells in each cell cycle phase. C) Drug response curve of siRNA-transfected H209 cells treated with AZD7762 (x-axis). The AUC of AZD7762 in siRNA-transfected cells is shown in the panel [All experiments were performed in quadruplicate. The values represent the mean ± SEM (Student’s *t-*test, **P*<0.05; ****P*<0.001)]


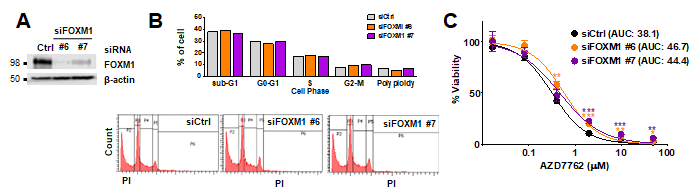


**Figure S6.** Pathway score bar plots of six EGFR-TKI resistance signatures according to four EGFR-TKI therapeutic groups. The numbers on each bar plot indicate the *P* values obtained using the Wilcoxon rank-sum test.


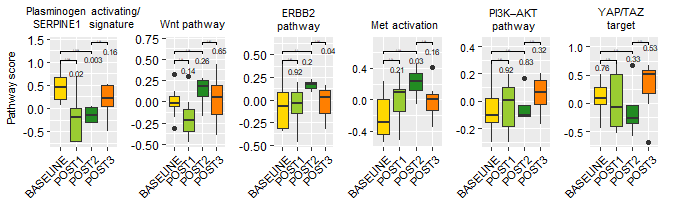


**Figure S7.** Drug candidates extracted from EGFR-TKI group PDCs (n=27) and extended EGFR PDCs (n=70). A) Volcano plots for EGFR-TKI groups of two datasets. The x-axis indicates the log_2_-fold change between drug responses, and the y-axis shows the log-scale adjusted *P* value. Red circles indicate sensitivity, whereas blue circle indicate resistance. B) Bar plots for etoposide and XAV939 AUC in both datasets. The X-axis indicates EGFR-TKI groups, and the Y-axis indicates AUC values. *P* values were obtained using the Wilcoxon rank-sum test.


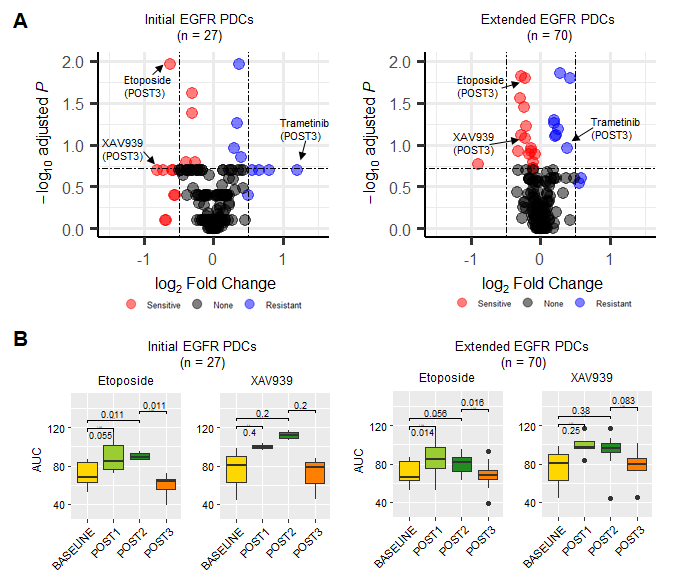

Supplement: Supplementary file 3 — Additional file 3: Fig. S1. Molecular subtype evaluation using six lung cancer cohorts (n = 1587). (A) Overall survival plots for each molecular subtype according to the subtype gene signature score. High and low groups were selected from the upper and lower quartiles. P-values and hazard ratios (HRs) were calculated using the log-rank test and Cox model. (B) HR forest plots for each subtype across six lung cancer cohorts. Fig. S2. Additional assessment of stemness scores for each molecular subtype. (A) A heatmap of average stemness scores according to molecular subtype. Stemness scores were assessed using GSVA from gene signatures: embryonic stem (ES) cell up-regulated genes (ES exp1, and ES exp2), and five transcription factors’ target genes as well as four PRC2 complex target signatures as control sets. (B) Boxplots of stemness scores according to subtypes. P-values were calculated by Wilcoxon rank-sum test to compare C3 and others. Fig. S3. Co-occurrent mutation case investigation. (A) Significant co-mutation pairs extracted using the Fisher’s exact test (recurrence > 5%). (B) The status of RB1/TP53 mutation and SCLC type. Fig. S4. Heatmap of DEGs between SCLC and NSCLC. The rows of the heatmap are the samples and the columns show the genes. Genes were extracted using limma (adjusted P < 0.005, |log2 FC| > 0.5) and the GSEA results (Table S3, Additional file 2) are summarized on the left. Fig. S5. Validation of the correlation of FOXM1 expression and AZD7762 sensitivity in H209 cells. (A) The protein levels of FOXM1 and β-actin in siRNA-transfected H209 cells (siFOXM1-#6, #7) analyzed using western blotting. Ctrl represents the control. H209 cells transfected with siRNAs were incubated for 48 h. (B) After incubation, the cells were analyzed using flow cytometry to evaluate the DNA content. Representative DNA content profiles from three independent experiments are shown. The graphs show the proportion of cells in each cell cycle phase. (C) Drug response curve of [file 13046_2023_2606_MOESM3_ESM.docx]
